# Nutritional knowledge and practice among antiretroviral therapy user adults in Bule Hora hospital, southern Oromia, Ethiopia

**DOI:** 10.3389/fnut.2024.1294233

**Published:** 2024-07-24

**Authors:** Habtamu Fekadu Gemede, Tamiru Yazew

**Affiliations:** ^1^Department of Food Technology and Nutrition, Wollega University, Nekemte, Ethiopia; ^2^Department of Public Health, College of Health Sciences, Salale University, Fitche, Ethiopia

**Keywords:** nutritional knowledge, adults, associated factors, dietary practice, antiretroviral therapy, Ethiopia

## Abstract

**Introduction:**

Nutrition is the necessary basis for life, health, and human development over the entire lifespan. Poor nutritional knowledge, poor nutritional practices, and malnutrition among HIV-positive adults can contribute to accelerating the progression of Human Immunodeficiency Virus (HIV)/Acquired Immunodeficiency Syndrome (AIDS) and related diseases. Therefore, this study aimed to assess the dietary knowledge, practices and associated factors of HIV-positive adults participating in antiretroviral therapy (ART) at Bule Hora Hospital, West Guji Zone, South Oromia, Ethiopia.

**Methods:**

A cross-sectional institutional study was conducted among 418 HIV-positive adults by systematic sampling technique. Semi-structured questionnaires were used for data collection and analyzed with SPSS version 21.0. Logistic regression analyses were used to identify factors associated with dependent variables using adjusted odds ratio (AOR), with 95% CI (confidence interval) at *p* < 0.05.

**Results:**

The result of this study showed that the prevalence of poor nutritional knowledge and poor nutritional practices among (HIV) positive adults was 74.9 and 69.1%, respectively. In the multivariate analysis, adult age (AOR = 2.37, 95% CI: 1.30, 4.32), marital status (AOR = 2.46, 95% CI: 1.29, 4, 69), educational level (AOR = 1.83, 95% CI: 1.01, 3.30) and occupational status (AOR = 0.55, 95% CI: 0.25, 0.94) were significantly associated with the nutritional knowledge. Educational level (AOR = 2.58, 95% CI: 1.48, 4.50), monthly income (AOR = 2.80, 95% CI: 1.68, 4.69), and adult occupational status (AOR = 0.48, 95% CI: 0.26, 0.89) were also significantly associated with the level of dietary practice.

**Conclusion:**

It was concluded that the respondents’ nutritional knowledge and practices in the city of Bule Hora were poor compared to other national findings. The identified factors related to nutritional knowledge and practices were educational level, monthly income, adult occupation, and marital status of respondents in the study area. Therefore, each concerned agency should address the above gaps in nutritional knowledge and practices of HIV-positive adults in the study area.

## 1 Introduction

Human immunodeficiency virus (HIV) continues to be a significant public health concern worldwide, resulting in the loss of 40.4 million lives to date, while transmission persists in every country across the globe (1). Globally, 15 nations are responsible for almost three-quarters of the total population living with HIV (2). It is of utmost importance to guarantee that individuals living with HIV in these countries are able to access essential HIV treatment services. Over the past 20 years, there has been undeniable evidence of significant achievements in reducing the negative impacts of HIV, such as morbidity, mortality, transmission, and stigma (3).

According to the Joint United Nations Program on HIV in 2016, there were approximately 36.7 million individuals living with HIV/ Acquired immunodeficiency syndrome (AIDS) worldwide (4). Out of this number, around 19.5 million people had access to antiretroviral therapy (ART). The African region continues to be the most heavily impacted, with 19.4 million individuals living with HIV/AIDS, including 11.4 million who have access to ART.

Ethiopia has one of the world’s highest rates of malnutrition and it is frequently observed in HIV-positive adults in Ethiopia who are in advanced stages of the disease, experiencing anemia, diarrhea, and have a Clusters of differentiation 4 (CD4) count below 200 cells/mm^3^ (5). The intertwining of malnutrition and human immunodeficiency virus (HIV) creates a relentless cycle that is further exacerbated in countries with low and middle-income levels. HIV/AIDS patients necessitate an additional 10% of energy when they are asymptomatic and 20–30% more when they experience symptoms, in contrast to uninfected individuals (6). Nevertheless, the presence of food insecurity and malnutrition adversely impacts the dietary consumption and quality life of these individuals (7). Malnutrition alone can have a detrimental impact on the cluster of differentiation-four (CD4+) T cells, resulting in an impaired B-cell response. Approximately 35 million people live with HIV/AIDS in the world (8).

Proper nutrition plays a crucial role in maintaining long-term health and overall well-being. Research indicates that individuals with HIV who consistently consume nutritious meals in appropriate portions are able to improve their tolerance to HIV medications, manage a healthy weight, and experience an enhanced sense of well-being (9). Good nutrition could be therefore improve the quality of life of people living with HIV/AIDS.

The 2011 Ethiopia Demographic and Health Survey (EDHS) showed that the urban prevalence of HIV/AIDS was 4.2% and in the rural it was 0.6% (10). The EDHS also further reported that HIV prevalence varies by region, ranging from 0.9% in Southern Nations, Nationalities, and Peoples’ Region (SNNPR) to 6.5% in Gambella regions of Ethiopia. For examples in 2005, Gambella, Addis Ababa, and Harari regions had the highest prevalence rates of 6.0, 4.7, and 3.5%, respectively (11). Conversely, the 2016 survey showed that Gambella regional state had the highest prevalence rate at 4.8%, followed by Addis Ababa at 3.4%.

Improper nutritional knowledge and practice management is a serious problem among people living with HIV/AIDS. Inadequate nutritional knowledge and practice of people with HIV infection lead to cause improper management of disease and lower immunological status as well as accelerates the progression of HIV/AIDS-related disease (12). In India, about 52% of HIV-positive patients had poor nutritional knowledge whereas 72% had poor nutritional practice (13). According to the study conducted in Ethiopia, about 25.8 and 3.2% of HIV-positive adults had poor nutritional knowledge and poor dietary practices, respectively (14).

Various factors influence the nutritional and dietary habits of individuals living with HIV. Among them, the occurrence of gastrointestinal symptoms, familiarity with the concept of good nutrition, and possessing adequate knowledge about nutrition were found to be influential factors in determining dietary practices (14). The study conducted in Ghana also reported that about 9.1% of the participants had inadequate knowledge about nutrition (15). In Nigeria, it was reported that 11.7% of women living with HIV/AIDS had low scores in nutritional knowledge (16). Another study conducted by Anand and Puri (17) revealed that 12.0% of people living with HIV from New Delhi, India had poor understanding of nutrition.

Despite progress made in the past 10 years, there is still a lack of representation of young adults in studies aimed at enhancing HIV prevention and treatment. Even though communities in the study area face disadvantages in comparison to those residing in other regions of Ethiopia, there is a lack of health and nutrition information available. Knowledge and practice of HIV-positive adults’ (>19 ages). Hence, the purpose of this research was to assess the nutritional knowledge, practices, and influencing factors in HIV-positive patients undergoing antiretroviral treatment at Bule Hora Hospital in Southern Oromia, Ethiopia.

## 2 Materials and methods

### 2.1 Description of the study area

The research was carried out at the Bule Hora Hospital Clinic, situated in the Oromia region of Ethiopia. This particular study is positioned in the West Guji Zonal Town, which is located to the south of Oromia and also south of the capital city, Addis Ababa. The distance between Bule Hora and Addis Ababa is approximately 470 km. The city of Bule Hora has a population of around 14,1,579 and is equipped with a health center and a hospital to cater to the healthcare needs of the residents and surrounding areas. In addition to serving the local population, this hospital also provides medical services to individuals from neighboring zones such as Gedao zone, including Gedab, Corso, and Chelelektu districts. Bule Hora Hospital currently has a total of 1,650 people living with HIV (PLHIV), all of whom are receiving antiretroviral therapy (ART) and being closely monitored.

### 2.2 Study design and period

A structured and pre-tested questionnaire was used to conduct an institutional cross-sectional study design, which aimed to evaluate the nutritional knowledge and practices of ART users who are HIV-positive adults aged over 19 years at Bule Hora Hospital. This study was carried out at Bule Hora Hospital in West Guji City, Oromia Regional State, Ethiopia, between September 23, 2019 and January 10, 2020.

### 2.3 Source and study population

All PLWHA, and ART user adults aged greater than 19 years old who were on follow-up in Bule Hora hospital were used a source population while those all PLWHA and ART user adults who were selected using systematic sampling method were used as study population.

### 2.4 Inclusion and exclusion criteria

Patients who had already started ART and were older than 19 years were included in the study area. Patients who are critically ill and unable to communicate, HIV cases who have not yet started ART, pregnant women, and non-voluntaries were excluded from the study.

### 2.5 Sample size determination

The minimum sample size of the study was calculated using the single population proportion formula with the following assumptions:


$$\;n=\frac{Z^{2}.p.q}{d^{2}}$$

Where: *q* = 1–P, *n* = a minimum number of sample size, *p* = 45% (0.45; proportion of undernutrition among adult HIV-positive individuals) in the study area from a similar survey (18), *d* = margin of error = 5%. Therefore, by considering 10% of non-response rate, the final sample size used for this study was 380.

### 2.6 Sampling procedures

The targeted sampling procedure was used to select the HIV-positive adult patients of Bule Hora Hospital to submit their responses to the questionnaire provided. Since the patients were arranged systematically, the systematic sampling method was used for this study. This sampling interval was explained using the formula: *K* = N/n, where: *K* = the sampling interval used to select every K^th^ item/subject from the sampling frame. *N* = total population size of patients with HIV in Bule Hora Hospital = 1,650, n = sample size (*n* = 418 patients). Therefore *K* = 1650/418 = ~4. Systematic sampling technique was used to select every 4th patient from the total sample frame of 1,650 HIV-positive adults who had follow up at the clinic to compose the 418 patients as the sample size. The data collection was carried out on a daily basis from September 2019 to January 2020 until the necessary sample for the study was obtained.

### 2.7 Study variables

The dependent variables of this study included dietary knowledge and practices of HIV-positive adults. The independent variables also included socioeconomic and demographic factors; gender, age, marital status, education level, family size, adult occupation, monthly income, ethnicity, place of residence, and religion.

### 2.8 Data collection method

The items of the questionnaire were developed from similar studies and adapted depending on the field of study. The details of nutritional knowledge and practices among adult ART users (>19 years) were collected by distributing structured questionnaires to respondents from the targeted study area. The data collection technique was performed using a semi-structured questionnaire to obtain all the required information. These questionnaires were developed in English and then translated into Afan Oromo, the local language, and back into English to ensure consistency using fluent individuals and pre-tested at nearby health center (Tore Health Center) on 5% of the total number of adults selected. The questionnaire determined information on socio-demographic characteristics (age, marital status, place of residence, level of education, family size, income, professional position, religion, and ethnicity); Nutritional knowledge, and practice of HIV-positive adults in the study area. Data collectors and supervisors received intensive training for two days on the purpose of the study, questionnaire, data collection methods, process of matching study participants, and ethics of the study during data collection.

To ensure the quality of the data, the English version of the questionnaire was carefully translated to the local language which was ‘Afaan Oromoo’ version and back to English by language translators to check for consistency. The data collectors received extensive training on how to use the questionnaires and information was collected under the close supervision of trained supervisors and principal investigators to obtain reliable and valid data. At the end of each day, the collected data were checked by principal investigator for completeness and consistency.

#### 2.8.1 Nutritional knowledge

Data on nutritional knowledge were collected using a semi-structured questionnaire from HIV-positive adults undergoing antiretroviral therapy in the study area. The questionnaires were designed on nutritional knowledge, containing 12 questions to categorize their knowledge into good and poor nutritional knowledge depending on their answer. Therefore, for every correct answer, the score is one (1) point and for every wrong answer, the score zero (0) point. Then, an individual could score a minimum of zero and a maximum of 12 points. Thus, the respondents who scored less than six (0–5.99) out of 12 questions had poor nutritional knowledge and those who scored greater than six (6–12) had good nutritional knowledge.

#### 2.8.2 Dietary practice

The dietary practice questionnaires were designed to assess the nutritional practices of HIV-positive adult patients (>19 years) attending an antiretroviral therapy (ART) clinic in the study area. There were 11 questions with positive and negative answers. Therefore, for every correct answer, the score is one (1) point and for every wrong answer, the score zero (0) point. Then, an individual could score a minimum of zero and a maximum of 11 points. Thus, the respondents who scored less than half (0–5.499) out of 11 questions had poor dietary practices and those patients who scored equal to or greater than half (5.5–11) had good dietary practices.

### 2.9 Ethical consideration

The ethical approval was obtained from the Research Review Board at Wollega University. A formal letter of cooperation was then requested from Wollega University to Bule Hora Hospital. Then a letter of support was written to the hospital’s ART Clinic office. Customer response was anonymous and the data collectors informed customers that they had the full right to withdraw from the study or to refuse to participate. A consent form was also attached to the questionnaire to obtain each individual’s permission.

### 2.10 Statistical analysis

The raw data collected through questionnaire survey, interview, and direct observation were first checked for completeness, coded, and entered into the computer using the Version 21 Statistical Package for Social Sciences (SPSS) for further analysis. Data on adult socio-demographic characteristics were summarized using descriptive statistics such as frequency, mean and percentage. In addition, Logistic regression analysis was also employed to see the association between dependent and independent variables at a *p* value less than 0.05. Finally, adjusted odds ratio (AOR) with a 95% confidence level was reported.

## 3 Result and discussion

### 3.1 Socio-demographic characteristics of the respondents

Four hundred and eighteen HIV-positive adults were sampled for this study and all completed the questionnaires with a 100% response rate. In this study, 45 and 55% of participants were male and female, respectively. Moreover, 35.9% of the respondents were married, 28.5% widowed, 23.7% divorced, and 12.0% were single (Table 1). In addition, about 36.8% of the study participants had no formal education. Concerning the residence, more than half participants were lived in urban area.

**Table 1:** Socio-demographic characteristics of respondents.

Variables	Category	Frequency	Percent
Sex	Male	188	45.0%
	Female	230	55.0%
Ages of adult	20–30	180	43.06%
	31–40	159	38.0%
	>40	79	18.9
	Mean age	33 ± 1.6	
Marital status	Single	50	12.0%
	Married	150	35.9%
	Divorced	99	23.7%
	Windowed	119	28.5%
Educational level	No formal education	154	36.8%
	Completed Primary	166	39.7%
	Tenth completed and above	98	23.4%
Place of residence	Urban	259	52.0%
	Rural	159	38.0%
Family size	≤ 3	253	60.5
	4–6	111	26.6
	≥7	54	12.9
Average monthly income	1,000–2000	168	40.2%
	2001–3,000	150	35.9%
	≥ 3,001	100	23.9%
Religion	Orthodox	125	29.9%
	Protestant	171	40.9%
	Wakefata	83	19.9%
	Muslim	39	9.3%
Adults occupation	Farmer/housewife	70	16.7%
	Merchant	106	25.4%
	Governmental employee	110	26.3%
	Daily laborers and others	132	31.6%
Ethnic group	Oromo	257	61.5%
	Gedao	97	23.2%
	Burji, Amhara and others	64	15.3%

### 3.2 Nutritional knowledge of respondents

According to the finding of this study, 53.1% of the respondents knew that fish is as a source of protein while 28.2% of the participants did not. A similar study was conducted in Nigeria, which shows that about 55.9% of adult HIV/AIDS patients undergoing ART considered fish as a protein source (12). This can be explained by the fact that the level of education and health information increases the nutritional knowledge of the respondents.

In this study, almost half of 49.5% respondents had information about high-protein foods that build and repair body tissues while 36.6% had no information. This study result is lower than the study conducted in Ethiopia, 73.7% (14) and Nigeria, 80.6% (16) and in Ethiopia, which show which show proteins are used to build and repair body tissues. This difference could be due to a lack of information, sample size and seasonality of food production in the study area.

According to the results of the study, more than half of 56.9%of the respondents knew carbohydrates and lipids as sources of energy and almost a third of 31.1% did not know. A similar study was conducted in the city of Bahir Dar, north-western Ethiopia which shows that 71.1% of respondents knew that carbohydrates and fats are energy-giving nutrients for adults who were suffered from HIV/AIDS (14).

Furthermore, the findings indicate that nearly 72.5% of the participants were aware of the significance of maintaining a well-balanced diet to prevent infections, whereas 17.2% of them lacked this knowledge (Table 2). The result of the study is comparable with the previous studies conducted in Nigeria, which shows that 70.1% of the respondents knew the importance of a balanced diet in preventing infection (16). The study finding is also similar with the study conducted in Ethiopia, which reports that about 77.2% of the adults on antiretroviral therapy knew that balance diet can be used as prevention of infection (14).

**Table 2:** Nutritional knowledge of respondents in the study area.

Variables/statements	Response			
		Yes, n (%)	No, n(%)	Do not know, n (%)
Knowledge about fish as sources of protein		222 (53.1)	118 (28.2)	78 (18.7)
Knowledge about the six component of food groups	F	193 (46.2)	162 (38.8)	63 (15.1)
Knowledge about food sources of carbohydrate		147 (35.2)	204 (48.8)	67 (16.0)
Knowledge about protein-rich food builds and repairs body tissues		207 (49.5)	153 (36.6)	58 (13.9)
Knowledge about carbohydrate and lipid as energy providers		238 (56.9)	130 (31.1)	50 (12.0)
Knowledge about balanced diet as preventing infection		303 (72.5)	72 (17.5)	43 (10.3)
Knowledge about the benefit of dietary diversity to HIV-positive patients		183 (43.8)	182 (43.5)	52 (12.4)
Knowledge about the source of iron		197 (47.1)	183 (43.8)	38 (9.1)
Knowledge about the source of vitamin- E		189 (45.2)	196 (46.9)	33 (7.9)
Knowledge about water as a nutrient		246 (58.9)	122 (29.2)	50 (12.0)
Knowledge about fruit and vegetable rich in vitamins and minerals		222 (53.1)	159 (38.0)	37 (8.9)
Knowledge about bananas as a control of diarrhea in HIV patients		236 (56.5)	165 (39.5)	17 (4.1)
Overall levels of nutritional knowledge	Good	105 (25.1%)		
	Poor	313 (74.9%)		

According to the finding of this study, almost half of the 47.1% respondents knew about the iron source and 43.8% of them did not. The finding of the study is higher than the study conducted in western Oromia region, Ethiopia, which shows that 19.3% of pregnant mothers knew the iron source (19). This could be because HIV-positive adults are in contact with health workers at least once a month to receive medication and nutrition related information to get iron rich foods and increase the production of red blood cells.

Moreover, the result of the study revealed that almost three-quarters of 74.9% of the respondents had poor nutritional knowledge and a quarter of 25.1%of the had good nutritional knowledge in the study area. The finding is relatively higher than that of the study conducted at Bahir Dar city, Ethiopia which shows that 21.7% of adults on ART had good knowledge of nutrition (14). A similar study conducted in Nigeria also found that 23.5% of women living with HIV/AIDS had a good knowledge of nutrition (16). However, this finding is lower than the study conducted in Uganda, which shows that 88% of women living with HIV/AIDS had a good nutritional knowledge (20). Similarly, about 70.9% of the people living with HIV in Ghan (15) and 67% of study subjects in Swaziland (21). had good nutritional knowledge, which are higher than the current study finding. The difference in results may be due to health and nutrition information, health services, socio-demographic factors, sample size and data collection period.

### 3.3 The nutritional practice of respondents in the study area

Table 3 shows that more than half 54.3% of the participants ate breakfast every day while 32.1% of them did not. Besides, the majority, 66.5% respondents ate their meal <3 times, while 33.5% of them ate greater than 3 times in the previous 24 h (Table 3). The finding of this study is supported by a study conducted in Uganda (20), which reports that 78.2% of women with HIV/AIDS eat their meals <3 times in the previous 24 h. The result of the study also revealed that almost three-quarters of 72.5% of the participants had milk and dairy products while 17.2% of them did not use milk and milk products in the study area. This study finding was relatively higher than the study conducted in Nigeria, which shows that the majority of respondents (66.3%) used milk and dairy products (16).

**Table 3:** Nutritional practice of respondents in the study area.

Variables		Frequency (n)	Percent (%)
Eating breakfast every day	Yes	227	54.3%
	No	134	32.1%
	Do not know	57	13.6%
Number of meals consumed in the preceding 24 h	< 3	278	66.5%
	≥3	140	33.5%
Eating milk and milk product	Yes	303	72.5%
	No	72	17.2%
	Do not know	43	10.3%
Preparing a balanced meal is not time consume	Yes	170	40.7%
	No	205	49.0%
	Do not know	43	10.3%
Washing fruit and vegetables before consumption	Yes	295	70.6%
	No	94	22.5%
	Do not know	29	6.9%
Taking medicine after a meal	Yes	345	82.5%
	No	51	12.2%
	Do not know	22	5.3%
Eating snacks between the main meal	Yes	207	49.5%
	No	194	46.4%
	Do not know	17	4.1%
Eating food during the period of illness	Yes	169	40.4%
	No	218	52.2%
	Do not know	31	7.4%
Drinking at least 8 glasses of water per day	Yes	329	77.8%
	No	60	14.8%
	Do not know	31	7.4%
Washing hands and utensils before & after the preparation of the food	Yes	304	72.7%
	No	76	18.2%
	Do not know	38	9.1%
Number of food groups consumed in the preceding 24 h	< 6	251	60.0%
	≥ 6	167	40.0%
Overall levels of nutritional practice	Good 129 30.9		
	Poor 289 69.1		

Regarding preparing a balanced diet, almost half of the 49.0% said preparing a balanced meal is time-consuming while more than a third of the 40.7% said it is not time-consuming. The result of the study, supported by the study conducted in Nigeria (16), which reported that 40.1% of women living with HIV/AIDS agreed that preparing a balanced meal is not time-consuming.

The results of this study revealed that the majority of 82.5% of the respondents took medication after a meal, while 12.2% did not take any medication after a meal. This finding is agreed with a study conducted in India (13), which shows that 95.3% of HIV-infected women on ART treatment take medication after a meal.

Similarly, this study identified that almost nearest to half 49.5% of participants did not use snacks. These results disagreed with research carried out in India, which suggested that 30 % (30%) of the women practiced consuming snacks/fruits between their main meals (13). This difference might be because of the time of study and lack of nutritional and health information.

The result of the study showed that more than half 52.2% of the participants did not practice eating food during the period of illness. Besides, more than three fourth 78.7% of respondents were drunk 8 glasses of water per day and 14.4% of them did not. The result of this study is similar to the study conducted in India which shows that more than three fourth (76%) of HIV-infected women with ART treatment practice 8 glasses of water per day (13).

Regarding washing hands and utensils before and after preparing food, almost three-quarters of participants 72.7% answered that they wash their hands and utensils, while very few of them 18.2% did not (Table 3).

The result of the study also revealed that the majority of 69.1% of the participants had poor dietary practices, while 30.9% of them had good dietary practices in the study area (Table 3). This result is relatively higher than the studies conducted in different regions of Ethiopia (14, 22–24). But the study finding is lower than the study conducted in Nigeria (16), which shows that 65.1% of women living with HIV/AIDS had good dietary practices. Another similar study was reported from the southern part of Ethiopia, which shows that about 60.1% of patients had inadequate dietary diversity (25).

Furthermore, this finding is lower than the study conducted in Nigeria (16), which shows that 65.1% of women living with HIV/AIDS had good nutritional practices. This study is similar with the study conducted in Switzerland, which shows that about 51% of pregnant and lactating women living with HIV/AIDS in had good nutritional practices (21). The study conducted in India showed that 28% of HIV-infected women had good nutritional practices (13).

### 3.4 Factors associated with the nutritional knowledge of respondents

In the multivariate analysis after controlling for possible confounders: adult age (AOR = 2.37, 95% CI: 1.30, 4.32), marital status (AOR = 2.46, 95% CI: 1.29, 4 0.69), an education level (AOR = 1.83, 95% CI: 1.01, 3.30) and occupation of adults (AOR = 0.49 and 0.55, 95% CI: 0.25, 0.94 and 0.31, 0.99) were factors significantly associated with the nutritional knowledge in the study area (Table 4).

**Table 4:** Factors associated with the nutritional knowledge of respondents in the study area.

Variables	Frequency (n)	Percent (%)	COR(95%CI)	*p* value	AOR(95%CI)	*p* value
**Age of adults**						
20–30	180	43.1	1		1	
31–40	159	38.0%	2.95 (2.04,4.26) *	0.001	1.46 (0.82,2.58)	0.23
>40	79	18.9%	4.48 (3.00,6.70)*	0.02	2.37 (1.30,4.32)*	0.01
**Marital status**						
Single	50	12.0%	1		1	
Married	150	35.9%	4.00 (2.00,8.00)*	0.001	1.55 (0.70,3.45)	0.34
Divorced	99	23.7%	2.85 (1.98,4.10)*	0.02	1.40 (0.84,2.34)	0.31
Widowed	119	28.5%	5.19 (3.04,8.86)*	0.023	2.46 (1.29,4.69)*	0.014
**Level of educational**						
No formal education	154	36.8%	1		1	
Completed primary	166	39.7%	4.13 (2.77,6.16)*	0.001	1.83 (1.01,3.30)*	0.02
Tenth complete & above	98	23.4%	2.77 (1.96,3.91) *	0.01	1.32 (0.77,2.28)	0.56
**Average monthly income**						
1,000–2000	168	40.2%	1		1	
2001–3,000	150	35.9%	3.20 (2.24,4.56)*	0.04	1.41 (0.82,2.43)	0.057
≥3,001	100	23.9%	3.55 (2.41,5.22)*	0.36	1.56 (0.88,2.75)	0.56
**Adults’ occupational status**						
Farmer/housewife only	70	15.6%	1		1	
Merchant	106	25.8%	2.04 (1.24,3.37)*	0.012	0.49 (0.25,0.94) *	0.001
Governmental employee	110	26.3%	2.31 (1.53,3.50)*	0.034	0.55 (0.31,0.99) *	0.002
Daily laborer	132	32.3%	3.40 (2.18, 5.31)*	0.001	0.91 (0.51,1.64)	0.061

*p < 0.05; statistically significant, 1; Reference, COR, Crude odds ratio; AOR, Adjusted odds ratio; ETB, Ethiopian Birr.

Accordingly, the age of the participants was one of the factors associated with the nutritional knowledge of HIV-positive adults in the study area. Thus, study participants whose age group was over (>40 years) were 2.37 times more likely to be knowledgeable than those whose age group was 15–20 years old (AOR = 2.37, 95% CI: 1.30, 4.32). This finding is supported by the studies conducted in Nigeria (16, 26). This could be because as the participants get older, so does their nutritional knowledge.

Moreover, HIV-positive widowed adults were 2.46 times more likely to be knowledgeable about nutrition than single adults (AOR = 2.46, 95% CI: 1.29, 4.69). The result of this study is similar to that of the study conducted in Nigeria (16). This could be due to a lack of partnerships to receive nutritional information and a lack of accessible education.

Additionally, the educational status of HIV-positive adults was significantly associated with nutritional knowledge in this study area. Respondents who completed elementary school were 1.83 times more likely to have knowledge than those who had no formal education (AOR = 1.83, 95% CI: 1.01, 3.30). This finding is supported by the study conducted in Nigeria, where women living with HIV/AIDS with at least secondary education were more likely to have nutritional knowledge than women with no education (16). Other studies conducted in Malaysia and Nigeria found that people with higher levels of education had better nutrition knowledge (26, 27).This could be because HIV-positive adults living in higher socioeconomic status had the opportunity to purchase and use various electronic devices important for improving nutrition education.

The study finding also reported that participants who were merchants and civil servants were 51 and 45% less likely to be nutritionally literate than those who were day laborers, respectively (AOR = 0.49 and 0.55, 95% CI: 0 0.25, 0.94 and 0.31, 0.99) field of study. This study was supported by research conducted Nigeria (12).

### 3.5 Factors associated with the nutritional practice of respondents

Although in the multivariate analysis, after controlling for possible confounders: educational level (AOR = 2.58, 95% CI: 1.48, 4.50), average monthly income (AOR = 2.80, 95% CI: 1.68, 4.69), and adult occupation (AOR = 0.48, 95% CI: 0.26, 0.89) were significantly associated with the level of dietary practice in the study area (Table 5).

**Table 5:** Factors associated with the nutritional practice of respondents in the study area.

Variables	Frequency (n)	Percent(%)	COR(95%CI)	*p* value	AOR(95%CI)	*p* value
**Educational level**						
Uneducated	154	36.8	1		1	
Completed Primary	166	39.7	3.53 (2.41, 5.17)*	0.01	2.58 (1.48, 4.50)*	0.001
Tenth complete & above	98	23.4	1.72 (1.26,2.36)*	0.21	1.17 (0.70, 1.95)	0.23
**Family size**						
≤3	253	60.5	1		1	
4–6	111	26.6	2.09 (1.60, 2.71)*	0.56	0.82 (0.50, 1.37)	0.45
> = 7	54	12.9	1.92 (1.30, 2.84)*	0.43	0.69 (0.38, 1.24)	0.79
**Monthly income in ETB**						
1,000–2000	168	40.2	1		1	
2001–3,000	150	35.9	3.10 (2.18, 4.41)*	0.001	2.80 (1.68, 4.69)*	0.01
≥3,001	100	23.9	2.19 (1.55, 3.09)*	0.04	1.89 (1.14, 3.14)*	0.02
**Occupational status of adults**						
Farmer or housewife	70	16.7	1		1	
Merchant	106	25.4	1.19 (0.74, 1.90)*	0.021	0.48 (0.26, 0.89)*	0.03
Governmental employed	110	26.3	2.79 (1.81, 4.29)*	0.76	1.28 (0.73, 2.26)	0.34
Daily laborers and others	132	31.6	2.24 (1.49, 3.35)	0.56	1.10 (0.64, 1.88)	0.12

*p < 0.05; statistically significant, 1; Reference, COR, Crude odds ratio; AOR, Adjusted odds ratio; ETB, Ethiopian Birr.

This finding was supported by the study conducted in Uganda, which shows that nutritional education and good nutritional practices were found to have a positive correlation. Their results were confirmed that well-structured dietary advice helps raise awareness of the importance of nutrition (20). Dietary advice not only improves food intake but also promotes dietary diversity (28). As nutritional knowledge increases, people living with HIV/AIDS tend to eat more meals than the traditional three meals in a day (29).

Another important factor influencing the dietary practices of HIV-positive adults was occupational status, according to this study. Respondents whose professional position is a merchant were 52% less likely to practice dietary practice (AOR = 0.48, 95% CI: 0.26, 0.89) in the study area are. This finding was supported by the study conducted in Swaziland (21). This could be because these HIV-positive adults with a commercial occupation may not have time for dietary practices (food intake), buying or selling things, and spending many hours outside the home to earn the income they may be earning in great tiredness. While HIV-positive adults who have only been housewives may have enough time to eat.

Monthly income, as a proxy indicator of socioeconomic status, is strongly associated with access to adequate food intake/nutrition security. The current study finding is agreed with studies conducted in different regions of Ethiopia (24, 25). This could be because a higher average monthly income plays an important role in sourcing the groceries and purchasing the nutritional diet and has also prompted respondents to discover new things in terms of nutritional practices.

## 4 Conclusion

The result of this study revealed that participants in the study area had poor nutritional knowledge and poor dietary practices compared to other studies results. Variables such as age, marital status, educational level, and average monthly income were factors associated with nutritional knowledge. Educational level, family size, average monthly income, and adult occupation were also factors significantly associated with dietary practice. Hence, it is imperative for healthcare professionals in hospitals to impart nutritional education to HIV-positive adults, thereby enhancing their understanding of nutrition. Specialized programs and interventions focusing on nutrition should be implemented to enhance the nutritional knowledge of HIV-positive adults in the region. Furthermore, it is essential for the organization to support ART patients, particularly HIV-positive adults, in improving their dietary habits and overall well-being. Each party involved should strive to bridge the existing gaps in nutritional knowledge, practices, and status among HIV-positive adults in the specified area.

## Data availability statement

The original contributions presented in the study are included in the article/supplementary material, further inquiries can be directed to the corresponding author.

## Ethics statement

The proposal was accepted and approved by the department, with ethical approval obtained from the Research Review Board at Wollega University. A formal letter of cooperation was then requested from Wollega University to BHH; then a letter of support was written to the hospital's ART Clinic office. Customer response was anonymous and the data collectors informed customers that they had the full right to withdraw from the study or to refuse to participate. A consent form was also attached to the questionnaire to obtain each individual's permission.

## Author contributions

HFG: Conceptualization, Formal analysis, Investigation, Methodology, Project administration, Resources, Software, Visualization, Writing original draft, Writing review & editing. TY: Conceptualization, Data curation, Formal analysis, Funding acquisition, Investigation, Methodology, Software, Supervision, Visualization, Writing original draft, Writing review & editing.
